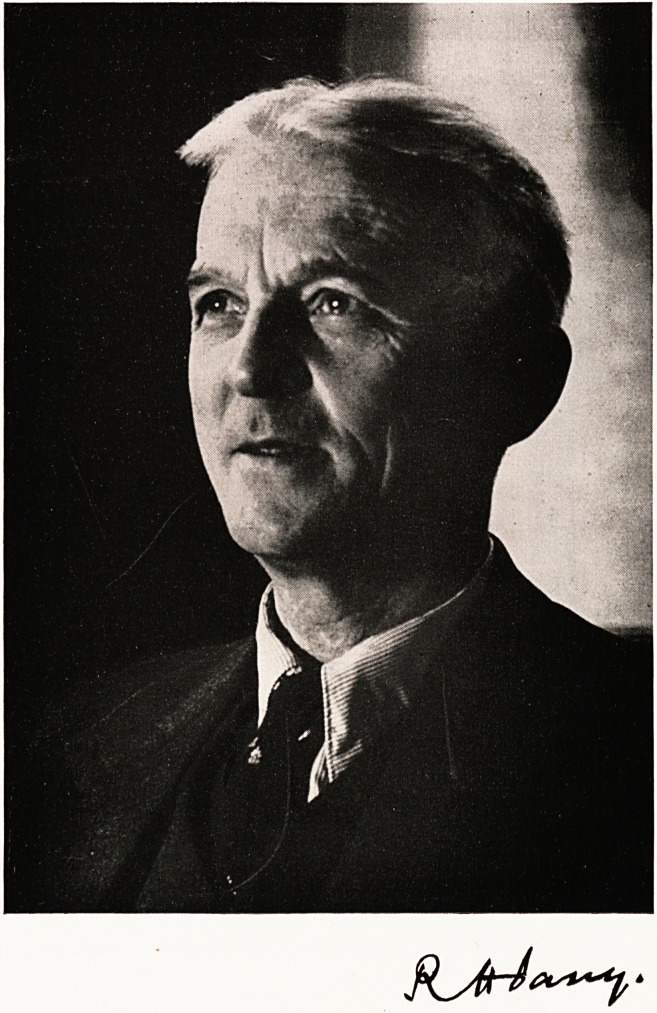# Dr. Robert Hughes Parry

**Published:** 1956-01

**Authors:** 


### *Editorial*

### DR. ROBERT HUGHES PARRY

Dr. Robert Hughes Parry, Medical Officer of Health for the City and County of Bristol and Professor of Preventive Medicine in the University of Bristol, retires in January, 1956.

Born in Caernarvonshire, North Wales, he received his early education at the University College of Wales, Aberystwyth. From there he proceeded to the Middlesex Hospital Medical School where, as a Lyell Scholar and Junior Broderip Scholar, he studied medicine gaining a " Conjoint " qualification in 1918. After a short spell as a Lieutenant in the R.A.F.M.S., he was appointed Casualty Surgical Officer at the Middlesex and then went on to spend three years as a Cancer Research Assistant. He graduated M.B., B.S. in 1921; in 1924 he gained his D.P.H., in 1933 M.D. (Lond.), in 1934 his M.R.C.P., and in 1943 was elected a Fellow of the Royal College of Physicians.

He first came to Bristol in 1924 as an Assistant Medical Officer of Health, but after only eleven months he returned to North Wales where, for the next three years, he practised in partnership with his wife, Dr. Joan Parry. In 1928 he returned to the City as Chief Assistant Medical Officer of Health and Assistant Port Medical Officer and two years later he was appointed as Medical Officer of Health, School Medical Officer and Port Medical Officer, succeeding Dr. R. Askins who had taken up an appointment in Rhodesia.

In 1933 the University appointed him Professor of Preventive Medicine. Since then there have been continuing and close relationships between the City Health Department and the University Department of Preventive Medicine to the mutual advantage of City and University. At the Preventive Medicine headquarters at Canynge Hall he built up a first-class chemical and bacteriological laboratory service and these facilities were offered to the general medical practitioners from an early date, thus anticipating by many years one of the most useful provisions of the National Health Service. In the last two years, under his direction, a Medical Statistical Section has been set up as a joint venture between the City and University.

During his tenure of office as Medical Officer of Health, the development of the personal health services in the City was rapid and intensive. Under his direction, the School Medical Service was co-ordinated with the Maternal and Child Welfare Service. In the thirties a big programme of hospital and clinic development was embarked upon. Thus Southmead Poor Law Institution was taken over for hospital purposes and was improved and extended; Ham Green Hospital and Sanatorium and Frenchay Park Sanatorium and Hospital were also extended and improved. Health clinics were built at Portway, Speedwell, Southmead and centrally and an integral part of the hospital and clinic service was the building up of a first-class specialist and consultant service. Dr. Parry's great experience and knowledge of hospital administration was recognized by his appointment as a member of the Advisory Council of the Nuffield Provincial Hospitals Trust. In the post-war years further clinics have been developed at Frenchay, Lawrence Weston, Brooklea, Claremont Street and Granby House and on Henbury and Hartcliffe Estates clinics are being built. It may be claimed, quite fairly, that Bristol has one of the finest clinic services in the British Isles. By offering clinic facilities to the general practitioners much has been done, too, to improve relationships between them and the health visitors.

His work has received world-wide recognition, thus adding distinction and honour to the City and the University. During the Second World War and again in 1951

he went to the U.S.A. on goodwill missions; there he visited many health departments and universities, making many good friends, some of whom have in turn paid Bristol the compliment of a return visit. In 1949 he spent four months in Greece as expert medical adviser to the American Mission. He has been an Honorary Fellow of the American Public Health Association for many years.

His colleagues in this country expressed their regard by electing him, in 1949, as President of the Society of Medical Officers of Health. Further honours included his appointment as Honorary Physician to the late King George VI and to Her Majesty, the Queen.

His personal charm and ready willingness to help those in need endeared him to his many students, colleagues and staff. He will undoubtedly be missed.

To round off a hard-working and brilliant career he will spend the first six months of 1956 as visiting Professor at Yale University, whereafter he will retire to his farm in Criccieth, North Wales. The foundations for his new " career " have been well laid and already he has been nominated as the 1958 Sheriff for his native county! He has the best wishes of all those who know him and have worked with him.

## Figures and Tables

**Figure f1:**